# Elemental and Proximate Compositions of Sesame Seeds and the Underlying Soil from Tsegede, Ethiopia

**DOI:** 10.1155/2022/1083196

**Published:** 2022-12-29

**Authors:** Fuad Seid, Bewketu Mehari

**Affiliations:** Department of Chemistry, College of Natural and Computational Sciences, University of Gondar, Gondar, Ethiopia

## Abstract

The proximate compositions and elemental contents of sesame (*Sesamum indicum*) seeds and the underlying soil from different cultivation areas of Tsegede, Ethiopia, were investigated. The ash, protein, fiber, fat, and carbohydrate contents of the sesame seeds were determined following standard methods. Essential major (Ca and Mg) and trace metals (Fe, Zn, Cu, Mn, and Ni) in the sesame seeds and the underlying soil were determined by using flame atomic absorption spectroscopy. The sesame seeds contained high levels of fat (52.9 ± 1.5%), followed by protein (23.5 ± 0.9%). The seeds contained 525 ± 1 and 453 ± 38 mg/kg of Mg and Ca, respectively. Iron was the most abundant (37.8 ± 1.4 mg/kg) of the trace metals, followed by Zn (14.6 ± 2.2 mg/kg) and Cu (7.26 ± 0.84). Manganese and Ni were found only in minute amounts. The concentrations of the trace metals varied significantly across the different cultivation areas. Similar to the sesame seeds, iron was found in higher amounts (212.6 ± 2.6 mg/kg) in the underlying soil followed by Zn and Cu, which were both 28.8 mg/kg. The amounts of the trace elements, Fe, Cu, and Zn found in the soils were about 2 to 6 times higher than that found in the sesame seeds. Whereas, the concentrations of Mg and Ca present in the seeds were comparable with that determined in the soils. Correlation analysis indicated that the Fe and Cu contents of the sesame seeds are negatively influenced by the amounts of Mg and Ca present in the soil. Ca in the seeds was also negatively associated with the Mg levels in soil.

## 1. Introduction

Sesame (*Sesamum indicum*) is a plant that belongs to the family *Pedaliaceae* and the genus *Sesamum*. The genus *Sesamum* consists of about thirty-six species, of which the most commonly cultivated is *Sesamum indicum* [1]. Sesame is cultivated for its edible seeds, which are relatively rich in oil and protein [2].

Sesame is one of the most important oil seed crops in the world [3]. The crop is cultivated both in the tropic and temperate zones of the world [4], where it is grown mostly for the edible oil extracted from its seeds. Sesame is a source of high quality edible oil with high preservative qualities [5]. The oil is also used in the production of perfumes, skin conditioners, and hair creams [3, 6].

Soil is the material that is found on the Earth's surface and consists of mineral, organic matter, water, and air [7]. The fertility of the soil has great contribution to sesame production and productivity. The fertility of a soil is determined by both its physical properties and its constituent chemicals that are essential for plant growth [8, 9]. In seed crops, like sesame seeds, nutrient imbalances may manifest themselves in quality characteristics of the fruit and its production [9].

Different soil types have different nutrient levels which are blended together in differing amounts. Soil properties like pH, moisture, and essential element content greatly influence plant growth [8, 10]. Essential elements are needed for both plants and animals as they form part of various tissues and act as catalysts in a wide range of metabolic processes [11].

Ethiopia ranks among the top six world producers of sesame seeds, and it is the second biggest export earner to the country after coffee [12]. Sesame is produced in many parts of Ethiopia, where Tsegede district stands as one of the main producers of sesame in the country.

The composition of the sesame seed is dependent mainly on genetic and environmental factors [13]. Different researchers have reported diverse results about the proximate composition of sesame seeds grown in different countries [14, 15]. This study aimed at determining the physicochemical characteristics of white sesame seeds, and the underlying soil, in Tsegede district, Amhara region, Ethiopia.

## 2. Experimental

### 2.1. Samples

**Figure 1:**
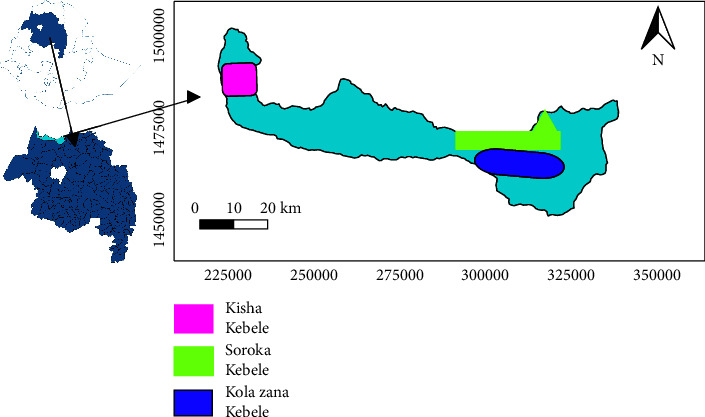
Map of Ethiopia, Amhara region, and Tsegede district depicting the studied sesame production areas.

Tsegede district is found in Amhara regional state, which is on the Northwest parts of Ethiopia between 147° 55′ 29.46″ N latitude and 37° 32′ 573″ E longitude (Figure 1). White sesame seeds and soil samples were collected from three different localities of Tsegede district (Soroka, Kisha, and Kola Zana) (Figure 1). The localities were selected based on their commercial importance. From each locality, three samples of sesame seed were collected from three different farmlands. Soil samples were collected at the depths of 15 cm from the sesame growing farmlands. From each farmland, five samples of soil were collected randomly, by walking diagonally and from rectangular angles, and mixed together thoroughly to produce a bulk sample. All samples were stored in polyethylene plastic bags.

### 2.2. Sample Preparation

The sesame seeds were cleaned manually to remove foreign matters, immature, and damaged seeds. The soil samples were air-dried left open in the air. The samples were then powdered and sieved through 0.5 mm mesh size and stored in polyethylene plastic bags at room temperature until analysis.

### 2.3. Determination of Sesame Seed Weight

A total of 2000 sesame seeds were counted manually and weighed using digital balance. The average weight of a sesame seed was calculated by dividing the measured weight by 2000.

### 2.4. Determination of Proximate Composition of Sesame

The proximate compositions (moisture, ash, fat, fiber, and protein) of the seeds were determined following standard methods (AOAC, 2010). Briefly, for moisture content determination, a 1 g sample was weighed in crucible and dried at 120°C for 6 h in oven. The moisture content was calculated as the loss in weight of the dried samples. The moisture content was used to calculate analytical results on the dry weight basis. The total ash content of samples was determined by the method of furnace incineration. For this, about 2 g of finely ground sample was converted to ash by heating in air at 600°C. The resulting ash was weighed and expressed as percentage to the weight of original sample. The crude fat content was determined after Soxhlet extraction of samples with 75 mL of hexane for 6 h at 40°C. At the end of the extraction, hexane was removed using rotavapor at 40°C, residue was dried at 100°C for 1 h, cooled, and weighed. For the determination of crude fiber content, 2 g of sample was extracted with 200 mL of 0.255 N H_2_SO_4_ for 30 min, filtered through muslin cloth and washed with boiling water. The residue was further extracted with 200 mL of 0.313 N NaOH for 30 min, filtered through muslin cloth, and washed successively with 25 mL of hot 1.25% H_2_SO_4_, twice with 50 mL of distilled water and 25 mL of alcohol. The residue obtained was dried at 130°C for 2 h, cooled in a desiccator, and weighed. Protein content was determined following the Kjeldahl method, while carbohydrate content was determined by subtracting the sum percentage of crude protein, fat, ash, moisture, and fiber from 100%.

### 2.5. Determination of Moisture Content of Soil

A 1 g soil sample was weighed, dried at 105°C for 24 h kept in an oven, cooled in a desiccator, and weighed again. The weight loss after drying was obtained by subtracting the weight of the dry sample from the original. The moisture content was used to calculate analytical results on the dry weight basis.

### 2.6. Determination of Soil pH

A 5 g of air-dried soil sample was weighed into a 100 mL beaker, 15 mL distilled water was added and the suspension was stirred vigorously for 20 min. The suspension was allowed to stand for about 30 minute for the suspended clay to settle out from the suspension. The pH value and temperature was then read and recorded immediately. The pH meter was calibrated with pH buffer 4, 8, and 11.

### 2.7. Sample Digestion

In order to analyze metals present in the sesame seed and soil samples, wet digestion method using HNO_3_ and HClO_4_ solutions in different proportions were tested and the one that consumed smaller reagent volume with minimum digestion time and temperature to produce clear and colorless solution was selected for the digestion of samples.

For sesame seed, a 1 g powder sample was first mixed with 6 mL of HNO_3_ (70%) and heated at 160°C for 3 h in a conical flask placed on a hot plate. Then after, 5 mL of HClO_4_ (30%) was added and heated until 2 mL of the volume remained. After cooling, 10 mL of distilled water was added, filtered through Whatman No. 42 filter paper, and diluted to 50 mL by adding distilled water.

For soil, a 1 g powdered sample was weighted into a conical flask and mixed with 6 mL HNO_3_ and 6 mL HClO_4_. The mixture was heated on a hot plate at 180°C for 3 h. After cooling, the digest was filtered through Whatman No.42 filter paper and diluted to 50 mL by adding distilled water. Each sample was digested in triplicates. Three blank solutions were also prepared following the same digestion procedure as the samples. During the measurement of Ca and Mg, lanthanum chloride was added to each sample and standard solution.

### 2.8. FAAS Determination of Elements

The concentrations of metallic elements in the digested samples were determined by using flame atomic absorption spectrophotometer (FAAS) (210VGP, Buck Scientific, USA) equipped with deuterium arc background corrector and air-acetylene flame at different operating conditions for each of the elements (Table 1).

The atomic absorption spectrometer was calibrated using seven point standard solutions, corresponding to each element, in the concentration (mg L^−1^) range of 0.10–2.00 for Mn, 0.50–4.00 for Fe, 0.10–2.00 for Ni, 0.50–3.00 for Cu, 0.10–1.60 for Zn, 0.50–10.00 for Ca, and 0.50–10.00 for Mg. The average triplicate readings were taken for each standard solution.

### 2.9. Method Validation

Accuracy, precision, and limits of detection were determined to assess the validity of the methods used for the digestion and analysis of the sesame seed and soil samples. The precision of the method was evaluated from the relative standard deviation of the results obtained from repeated measurements made on a given sample. Limit of detection of the method was calculated as three times the standard deviation of the blank signals divided by the slope of the calibration equation. Accuracy was determined by spiking the samples with known concentrations of standard solutions. For this, a 1 g powdered sample of sesame seed or soil was fortified with a standard solution at a concentration level corresponding to 100% of the average measured value for each element and subjected to the digestion and analysis procedure.

### 2.10. Statistical Analysis

One-way analysis of variance was used to test the effect of the growing region on the mean concentrations of the different chemical constituents determined. Statistical analyses of the data were carried out using Excel (Microsoft Excel, 2007). Differences were considered significant when *α* < 0.05.

## 3. Results and Discussion

### 3.1. Seed Weight

The mean seed weights of sesame were 5.21 ± 0.02, 5.89 ± 0.05 and 6.25 ± 0.02 g/2000 seeds from Kisha, Kola Zana, and Soroka, respectively. The measured seed weights are in the range of 4‒7 g/2000 seeds reported by Al-Kahtani [16] from Saudi Arabia. The weight of a sesame seed is, however, significantly different across the three studied production areas, indicating the influence of the growing environment on the seed weight of sesame.

### 3.2. Proximate Composition

The sesame seeds were rich in fat (average 52.86% dry weight), with considerable amount of protein (average 23.52% dry weight) followed by carbohydrate (average 14.51% dry weight) (Table 2). Ash and fiber were present in lower and comparable proportions, 4.84 and 4.27%, respectively, in the seeds.

The moisture contents of the sesame seeds varied in the range of 5.43–5.81%, with no statistically significant difference, across the three production areas. On the other hand, the levels of the determined dietary components of the sesame seeds varied significantly with production origin. The ash contents of the sesame seeds varied in the range 4.38–5.48%, with significantly higher amount of ash found in seeds from Kola Zana (5.48%) than those from the other areas. Crude fiber was found in significantly higher amounts in seeds from Kisha (4.97%) than that from the other areas (3.88–3.95%). Sesame seeds from Soroka contained significantly higher levels of crude fat and protein while lower carbohydrate than seeds from the other areas.

The average concentrations of the dietary components determined in the sesame seed samples from the three areas were compared with some reported values for white sesame seeds from different countries (Table 3). For the sake of comparison data obtained from the sesame seed samples have been recalculated to the fresh weight basis using the determined moisture content values. The moisture contents of the sesame seed samples are comparable with those seeds from Congo-Brazzaville (5.7%) [15] and Nigeria (5.2%) [17], while higher than those from Turkey (4.40%) [18] and China (4.71%) [19]. On the other hand, the determined values are lower than that (6.91%) reported by Okoronkwo et al. [20]. The moisture contents of the samples are all below 6% that has been indicated as good condition for sesame seeds at harvesting [21]. Lower moisture content has also been indicated as beneficial to the quality and shelf life of sesame seeds [22].

The sesame seed samples contained significantly higher amounts of ash and crude fiber than seeds from Congo-Brazzaville and Nigeria, while comparable in ash content with seeds from Turkey and China. Ash content is an indication of mineral elements that are present in the sesame seeds, while fiber in diet is important as it helps to maintain human health by reducing cholesterol level in the body [23].

The seed samples contained about twice of crude protein and half of carbohydrate than seeds from Nigeria. Whereas, the crude protein and carbohydrate contents of the seeds are comparable with those from Turkey and China.

### 3.3. Elemental Composition of Sesame Seeds

#### 3.3.1. Analytical Characteristics of the Method

The reliability of the optimized digestion procedure used in the determination of essential metals in the samples using FAAS was evaluated with respect to analytical figures of merit. The correlation coefficients (*r*^2^) obtained for the calibration curves were in the range of 0.9966–0.9997 (Table 4), indicating a good linear relationship between concentration and absorbance. The limit of detection of the method ranged 0.09–0.38 mg/kg across the different elements.

The average percentage recoveries for the studied metals in the sesame seed sample spikes ranged between 94.1 and 112.4%. The precision of the method, which was expressed as the relative standard deviation of three replicate measurements made on spiked samples, ranged from the 0.24 to 14.95% across the different elements. Both the accuracy and precision of the method were good enough to allow the quantitative determination of the nutrient elements in the samples.

#### 3.3.2. Concentration of Elements in Sesame Seeds

The overall mean concentration of the major elements Mg and Ca found in the sesame seeds were 525 and 453 mg/kg dry weight, respectively (Table 5). These values are comparable with the 579.53 mg/kg Mg and 415.38 mg/kg Ca reported for sesame seeds from Congo-Brazzaville by Nzikou et al. [15].

Among the determined trace metals in the sesame seeds, the most abundant was Fe (average 37.8 mg/kg) followed by Zn (average 14.6 mg/kg) and Cu (average 7.26 mg/kg). The amount of Fe found in the sesame seed samples falls within the range of 35.20–43.10 mg/kg reported by Gebrekidan and Desta [24]. The determined concentration of Zn also falls within the range 13.92–28.49 mg/kg reported for sesame seeds from Turkey by Cemal [25]. On the other hand, the amount of Cu found in the samples is lower than that (13.5 mg/kg) reported by Obiajunwa et al. [26] for sesame seeds from Nigeria. The elements Mn and Ni were found only in minute amounts in the sesame seed samples.

The concentration of the trace metals determined in the sesame seeds varied significantly with the cultivation regions. Sesame seeds from Kola Zana contained higher levels of Fe and Cu than seeds from the other cultivation areas. On the other hand, sesame seeds from Kisha contained lower levels of Zn and Mn than seeds from the other cultivation areas. This may be due to differences in the environmental growing conditions. The elemental composition of a plant is generally a reflection of the elemental composition of the soil in which the plant was cultivated [27]. However, in this study no significant difference was found in the concentrations of Fe and Cu among soils from the different cultivation areas. Hence, the observed differences may be due to other environmental factors, such as air temperature and rainfall. The accumulation of an element within a plant depends on environmental conditions. Plants of the same species that are grown in different geographical locations may exhibit varying elemental profiles as a result of the environmental conditions [28].

The elements Fe, Zn, Cu, and Mn are trace essential elements for humans. The elements are micronutrients that are required in minute quantities to play vital role in the normal functioning of various physiological and metabolic processes [29]. Zinc is an essential trace element required for the functioning of many enzymes, supporting the immune system, and excess Zn is toxic and interferes with the metabolism of other minerals in the body, particularly Fe and Cu [30]. Copper is essential for a variety of enzymes and also involved in the functioning of the nervous system [31]. Iron plays an important role in the function of hemoglobin, and iron deficiency causes anemia and infertility, while its excess can damage tissues of the kidneys, heart, and lungs [32].

#### 3.3.3. Moisture Content and pH of the Sesame Growing Soil

The pH of the soil samples were in slightly acidic range with values of 5.26, 5.42, and 5.58 at Kola Zana, Soroka, and Kisha, respectively. There was no statistically significant difference among the cultivation areas with respect to pH. The pH of the soil samples are within the range of 5‒8 that has been indicated as best condition for growing sesame [33]. The moisture content of the soil samples were 7.9% at Soroka and 6.7% at both Kisha and Kola Zana.

### 3.4. Elemental Composition of the Sesame Growing Soil

#### 3.4.1. Analytical Characteristics of the Method

The reliability of the optimized digestion procedure used in the determination of essential metals in the samples using FAAS was evaluated with respect to analytical figures of merit. The correlation coefficients (*r*^2^) obtained for the calibration curves were in the range of 0.9985–0.9991 (Table 6), indicating a good linear relationship between concentration and absorbance. The limit of detection of the method ranged 0.087–0.377 mg/kg across the different elements.

The average percentage recoveries for the studied metals in the soil sample spikes ranged between 89.7 and 94.2%. The precision of the method, which was expressed as the relative standard deviation of three replicate measurements made on spiked samples, ranged from the 0.50 to 12% across the different elements. Both the accuracy and precision of the method were good enough to allow the quantitative determination of the nutrient elements in the samples.

#### 3.4.2. Concentration of Elements in Soil

The average determined concentration of Mg and Ca across the different cultivation areas were 507.6 and 418.0 mg/kg, respectively (Table 7). Similar to the sesame samples, the most abundant trace metal was Fe, but with the average concentration of 212.6 mg/kg across the different cultivation areas. Copper and Zn were found in equivalent proportions, with average concentrations of 28.8 mg/kg, across the different cultivation areas. There was no statistically significant difference in the concentration of Fe and Cu among soils from the different cultivation areas. On the other hand, significantly higher concentration of Zn was found in soil samples from Soroka than that from the other areas.

Pearson correlation analysis was performed between the elemental compositions of sesame seeds and the underlying soil (Table 8). The analysis was applied to assess the relationship between the concentration of a metal present in the soil with that in the sesame seeds, as well as to assess whether a metal present in soil facilitate or interfere with the uptake of another metal. Considering strong correlations, correlation coefficient ≥ |0.9|, increased concentration of Mg in sesame seeds is associated with decreased concentration of Fe and Cu in soil. The level of Fe and Cu in the sesame seeds is affected negatively by the presence of higher amounts of Mg and Ca in soil. The amount of Ca in sesame seeds decreases with increasing concentration of Mg in soil.

## 4. Conclusion

The proximate composition and elemental content of sesame seeds varies with cultivation region in Tsegede district, Ethiopia. The seeds are rich in fat followed by protein and carbohydrate. The seeds also contain considerable amounts of the essential trace metals Fe, Zn, and Cu. The concentrations of these elements in the sesame seeds varied significantly with the cultivation regions. Whereas, no significant difference in the concentration of the elements, except Zn, among soils from the different cultivation areas. The levels of Fe and Cu in the sesame seeds are affected strongly and negatively by the presence of higher amounts of Mg and Ca in the soil.

## Figures and Tables

**Table 1:** Instrument operating conditions used for the determination of metallic elements using flame atomic absorption spectroscopy.

Element	Wavelength (nm)	Slit width (nm)	Lamp current (MA)	Energy (J)
Ca	422.7	0.7	2.0	3.914
Mn	279.5	0.7	3.0	4.080
Cu	324.8	0.7	1.5	3.786
Zn	213.9	0.7	2.0	3.101
Fe	248.3	0.2	7.0	3.085
Ni	232	0.2	7.0	3.151
Mg	285.5	0.7	1.0	3.939

**Table 2:** Proximate composition (%w/w dry weight) of sesame seeds from three different cultivation areas of Tsegede, Ethiopia

Sample area	Crude ash	Crude fiber	Crude fat	Crude protein	Carbohydrate
Soroka	4.38 ± 0.29	3.95 ± 0.13	54.78 ± 0.84	24.45 ± 0.38	12.45 ± 0.36
Kola Zana	5.48 ± 0.20	3.88 ± 0.26	52.11 ± 0.54	23.64 ± 0.64	14.90 ± 0.57
Kisha	4.65 ± 0.12	4.97 ± 0.03	51.69 ± 0.08	22.49 ± 0.52	16.20 ± 0.57
Overall mean	4.84 ± 0.53	4.27 ± 0.59	52.86 ± 1.52	23.52 ± 0.89	14.51 ± 1.82

**Table 3:** Comparison of the proximate composition (%w/w dry weight) of white sesame seeds from different countries.

Characteristics	Sample origin				
	Congo	Nigeria	Turkey	China	Ethiopia^*∗*^
Moisture	5.7 ± 0.24	5.2 ± 0.35	4.40 ± 0.05	4.71 ± 1.34	5.67 ± 0.21
Ash	3.7 ± 0.92	3.6 ± 0.02	4.41 ± 0.05	4.32 ± 2.41`	4.57 ± 0.50
Crude fiber	3.2 ± 0.22	2.6 ± 0.02	—	—	4.03 ± 0.56
Crude fat	54 ± 0.16	48.0 ± 0.55	54.26 ± 0.99	52.61 ± 0.87	49.86 ± 1.43
Crude protein	20 ± 0.12	11.6 ± 0.15	21.0 ± 0.09	22.20 ± 0.67	22.19 ± 0.84
Carbohydrate	13.4	29.0 ± 0.25	—	15.54 ± 0.74	13.69 ± 1.72
Ref.	[15]	[17]	[18]	[19]	This study

^*∗*^Average values across the three production areas studied. Data have been recalculated to the fresh weight basis using the determined moisture content values.

**Table 4:** Concentrations of standard solutions, coefficient of determination (*r*^2^), limit of detection (LOD), recovery, and the associated relative standard deviation (RSD) values of the method used for the determination of essential metallic elements in the sesame seed samples.

Element	Concentration (mg/L)	*r* ^2^	LOD (mg/kg)	%Recovery	RSD
Fe	0.5, 1, 1.5, 2, 3, 4	0.9966	0.17	96.4	11.6
Zn	0.1, 0.4, 0.8, 1.2, 1.6	0.9984	0.30	96.9	3.79
Ni	0.1, 0.2, 0.5, 1, 2	0.9990	0.09	96.9	14.9
Cu	0.5, 1, 1.5, 2, 3	0.9989	0.15	112.4	9.04
Mn	0.1, 0.2, 0.5, 1, 2	0.9995	0.09	95.0	14.8
Ca	0.5, 1, 2, 4, 8, 10	0.9997	0.26	94.1	8.41
Mg	0.5, 1, 2, 4, 8, 10	0.9988	0.31	97.5	0.24

**Table 5:** Concentration of metals (mg/kg, dry weight) in sesame seeds from three different cultivation areas of Tsegede, Ethiopia.

Sample area	Fe	Zn	Ni	Cu	Mn	Ca	Mg
Soroka	37.0 ± 1.1	15.8 ± 0.9	1.86 ± 0.77	6.52 ± 1.56	2.31 ± 0.16	453 ± 40	524 ± 2
Kola Zana	39.4 ± 5.1	16.0 ± 0.9	1.86 ± 1.07	8.17 ± 1.88	2.63 ± 0.38	492 ± 37	526 ± 1
Kisha	36.9 ± 5.1	12.1 ± 1.1	1.59 ± 0.49	7.08 ± 0.75	1.96 ± 0.16	415 ± 52	526 ± 4
Overall mean	37.8 ± 1.4	14.6 ± 2.2	1.77 ± 0.16	7.26 ± 0.84	2.30 ± 0.34	453 ± 38	525 ± 1

**Table 6:** Concentration of standard solutions, coefficient of determination (*r*^2^), limit of detection (LOD), recovery, and the associated relative standard deviation (RSD) values of the method used for the determination of metallic elements in the soil samples.

Elements	Concentration (mg/L)	*r* ^2^	LOD (mg/kg)	%Recovery	RSD
Zn	0.1, 0.4, 0.8, 1.2, 1.6	0.9985	0.26	90.7 ± 15	6.7
Cu	0.5, 1, 1.5, 2, 3	0.9986	0.23	94.0 ± 1.8	0.5
Fe	0.5, 1, 1.5, 2, 3, 4	0.9986	0.15	89.7 ± 5.3	1.2
Ca	0.5, 1, 2, 4, 8, 10	0.9991	0.09	90.6 ± 2.6	12.0
Mg	0.5, 1, 2, 4, 8, 10	0.9987	0.38	94.2 ± 9.8	2.7

**Table 7:** Concentration (mg/kg, dry weight) of metals in soil samples from three sesame growing areas of Tsegede, Ethiopia.

Sample area	Fe	Zn	Cu	Mg	Ca
Soroka	215.5 ± 0.4	31.0 ± 1.6	28.9 ± 2.2	511.6 ± 8.8	456.8 ± 4.3
Kola Zana	212.0 ± 1.6	27.5 ± 1.6	28.9 ± 0.5	492.1 ± 6.1	362.6 ± 7.9
Kisha	210.4 ± 2.4	27.8 ± 2.8	28.7 ± 2.4	518.7 ± 27	435.4 ± 70
Overall mean	212.6 ± 2.6	28.8 ± 1.9	28.8 ± 0.1	507.6 ± 14	418.0 ± 49

**Table 8:** Pearson correlation coefficients between the elemental compositions of sesame seeds and soil.

Sesame composition	Soil composition				
	Zn	Fe	Cu	Ca	Mg
Mg	−0.93592	−0.99959	−0.61262	−0.44088	0.015207
Ca	−0.09031	0.293697	0.924958	−0.74779	−0.96784
Fe	−0.54498	−0.18674	0.640377	−0.97131	−0.97329
Cu	−0.81029	−0.52789	0.320935	−0.9918	−0.82538
Zn	0.384603	0.705621	0.995459	−0.35182	−0.73881

## Data Availability

All data generated during this study are included in the manuscript.
